# Probiotic *Shewanella putrefaciens* (SpPdp11) as a Fish Health Modulator: A Review

**DOI:** 10.3390/microorganisms8121990

**Published:** 2020-12-14

**Authors:** María Cámara-Ruiz, María Carmen Balebona, Miguel Ángel Moriñigo, María Ángeles Esteban

**Affiliations:** 1 Immunobiology for Aquaculture Group, Department of Cell Biology and Histology, Faculty of Biology, Campus Regional de Excelencia Internacional “Campus Mare Nostrum”, University of Murcia, 30100 Murcia, Spain; Maria.camara1@um.es; 2 Group of Prophylaxis and Biocontrol of Fish Diseases, Department of Microbiology, Campus de Teatinos s/n, University of Malaga, 29071 Málaga, Spain; balebona@uma.es (M.C.B.); morinigo@uma.es (M.Á.M.)

**Keywords:** gilthead seabream, Senegalese sole, SpPdp11, sustainable aquaculture, probiotics

## Abstract

Aquaculture is considered one of the largest food production sectors in the world. Probiotics have long been considered as a beneficial tool in this industry since these microorganisms improve the welfare of different fish species by modulating several physiological functions, such as metabolism, digestion, immune response, stress tolerance, and disease resistance, among others. SpPdp11, a probiotic isolated from the skin of healthy gilthead seabream, has been the center of attention in a good number of studies since its discovery. The purpose of this paper is to summarize, comment, and discuss the current knowledge related to the effects of SpPdp11 in two commercially important fish species in aquaculture (gilthead seabream and Senegalese sole). Furthermore, some considerations for future studies are also indicated.

## 1. Introduction

The world will face an enormous challenge when it comes to providing food for an expected 9 billion people by the middle of the twenty-first century, in (what is expected to be) a complicated context of climate change, environmental degradation, and economic instability. Aquaculture continues to grow faster than any other major food production sector and approximately represents 50% of sea products for human consumption, providing a source of healthy protein at an affordable price [1].

The probiotics under study in this review was investigated, to present, in two farmed marine fish species, gilthead seabream (*Sparus aurata*) and Senegalese sole (*Solea senegalensis*). The Gilthead seabream aquaculture industry has grown significantly since the 1990s and represents one of the most important species cultured in the Mediterranean area. On the other hand, Senegalese sole has been a promising species for the diversification of aquaculture since the 1980s; however, technical and disease problems are responsible for the main setbacks in the development of the sole farming industry [2,3]. Similar to other animal farming systems in which animals are raised in high numbers, the intensive farming of fish can potentially increase the risk of disease outbreaks. In particular, gilthead seabream can be affected by a range of viral and bacterial pathogens, including lymphocystis disease virus (LCDV) and bacterial diseases, such as vibriosis. They can also be affected by parasites, such as *Sparicotyle chrysophrii*, *Amyloodinium ocelatum*, and *Cryptocaryon irritans*. Regarding Senegalese sole, one of the most serious problems is the outbreak of infectious diseases associated to pseudotuberculosis, which is caused by *Photobacterium damselae* subsp. *piscicida* [4,5,6].

Control of pathogens has routinely been achieved by the administration of antimicrobial agents, such as antibiotics, in fish farms. The excessive use of these products has led to the emergence of antibiotic-resistant bacteria, which has become a risk to the success of the treatments, to human health, and to the environment. Because of this, strict regulations have been established to ban or minimize their application in aquaculture [7,8]. Modern aquaculture demands alternative practices that help maintain high animal welfare, as well as a healthy environment. The development of functional feeds is becoming one of the main topics in the aquaculture industry, trying to develop balanced and eco-friendly diets with feed additives to improve overall fish health. Feed additives, such as probiotics, prebiotics, and immune-stimulants, have earned the attention of many researchers in the last decades [9,10].

Probiotics were defined as live microbial feed supplement, which beneficially affect the host animal by improving its microbial balance [11]. Then, probiotics were defined by the World Health Organization as “live micro-organisms which, when administered in adequate amounts, confer a health benefit on the host, apart from the traditional nutritional effect”. However, this definition is constantly being modified with health-promoting properties that are being studied. A more recent and modified definition for probiotics in aquaculture was proposed by Merrifield et al. [12] as “a probiotic organism can be regarded as alive, dead or component of a microbial cell, which is administered via the feed or to the rearing water, benefiting the host by improving disease resistance, health status, growth performance, feed utilization, stress response or general vigour, which is achieved at least in part via improving the host’s microbial balance or the microbial balance of the ambient environment”. Probiotics, which are widely used in aquaculture, include different kinds of bacteria, microalgae, and yeast cells [13]. Taking into account this probiotic definition, these products can be useful in healthy fish specimens in order to optimize some physiological functions or to reduce the possible risk of developing certain diseases, as well as in ill or infected fish to help overcome such pathologies.

Currently, there are several commercially available probiotics. The most common probiotics used in aquaculture are *Lactobacillus* sp., *Bacillus* sp., *Bifidobacterium* sp., *Vibrio* sp., *Saccharomyces* sp., and *Enterococcus* sp., among others [14]. A bacterium isolated from the skin of healthy gilthead seabream, *Shewanella putrefaciens* (known as Pdp11 or more recently as SpPdp11), has been used in different studies to establish its application as a probiotic in the culture of the aforementioned-farmed fish species. In this review, we focus firstly on the characterization of this microorganism, taking a special interest in its characteristics, which make it a good candidate to be considered as a probiotic for farmed fishes. Then, we continue with in vivo trials and their major outcomes performed until the present, and to conclude, we sum up all findings made so far, and suggest other approaches for future research with this microorganism.

## 2. Probiotic Administration Routes

**Figure 1:**
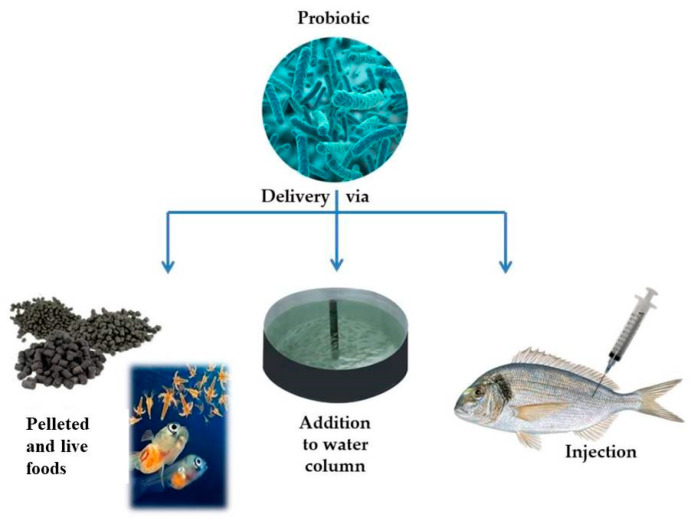
Different probiotic administration routes in aquaculture systems.

There are several methods when administering probiotics in aquaculture systems, delivery via injection, direct addition to the water column, delivery via feeding of supplemented live food with probiotics, and delivery via feeding on supplemented pellet food with probiotics (Figure 1) [15].

Delivery via injection, which is not applicable for larvae, results in a high level of stress for the animals, which is not recommendable, and it is very expensive (time consuming and it needs some experience for handling fish). Regarding direct addition to the water column, it is the only method that is applicable for all ages of fish. Furthermore, this method has two main advantages: the ability to control the quality of water by bioremediation and the bio-control of pathogens [16,17]. In fact, the combination of probiotic administration through water and enriched live feed has been strongly recommended as the most appropriate way to apply probiotics in larviculture [15]. However, this method cannot be applied when fish are being reared in open sea cages. Finally, the administration via dry feed definitely has limitations during early larval stages because of immature digestive tracts of fish in that stage of development. In conclusion, the probiotic administration method should be carefully selected based on the age, size, species of fish, and the aquaculture system rearing the animals.

## 3. SpPdp11 Characterization

A common and classic way to select a potential probiotic is to perform in vitro antagonism tests based on the production of inhibitory compounds or competition for nutrients with bacterial pathogens [18]. Furthermore, the adhesion to host surfaces and adhesive interactions with pathogens is usually also taken into account for the selection of probiotics [18,19,20]. These properties are very important when choosing a probiotic since they should survive the passage through the digestive system (in other words, both gastric acid and bile to reach the posterior intestine), and they should be able to attach themselves to the intestinal epithelia and colonize. Furthermore, probiotics should be safe, non-pathogenic, non-toxic, and capable of exerting a beneficial effect on the host (e.g., anti-inflammatory, anti-mutagenic, immunostimulatory). 

Studies on SpPdp11 probiotic started when several bacterial strains were isolated from healthy gilthead seabream and Senegalese sole. Of all strains, four were selected and evaluated based on their adhesive ability to skin and intestinal mucus of Senegalese sole, and their antagonistic effect against *Vibrio harveyi* and *P. damselae* subsp. *piscicida* [21]. Regarding adhesion capacity, SpPdp11 improved this feature in sole mucus, demonstrating positive characteristics of this probiotic. Moreover, SpPdp11 showed not only a higher adhesion capacity to sole mucus, but also antagonistic activity against them because it was able to reduce the attachment to skin and intestinal mucus of these pathogenic bacteria [22,23]. Afterwards, the in vivo potential of SpPdp11 was assessed in sole by oral administration followed by a challenge with *V. harveyi* being the mortality of fish recorded for 20 days after challenge. The mortality in fish receiving the diet supplemented with SpPdp11 was significantly lower in comparison with the fish, which received a control diet [22]. These positive results encouraged researchers to continue working on the characterization of the properties of this beneficial bacterium.

SpPdp11 also showed ability to interfere with pathogenic organisms, such as *Vibrio anguillarum,* when it was evaluated in gilthead seabream [23]. Again, SpPdp11 showed ability to adhere to skin, gill, and intestinal mucus, and had antagonistic effect against the pathogen. Furthermore, it specifically interfered with the attachment of *Listonella anguillarum* to gilthead seabream skin mucus. After these interesting in vitro results, SpPdp11 was chosen for an in vivo trial. Gilthead seabream were fed with SpPdp11 and challenged with *L. anguillarum*. Similar to the results obtained in sole, SpPdp11 was able to reduce the mortality in gilthead seabream infected with *L. anguillarum* [23].

After these initial findings, several studies have addressed potential effects of SpPdp11 administration in these two fish species, and even more concretely, a recent study has shed light into its genomic characteristics using an automatized workflow called TarSynFlow (Targeted SyntenyWorkflow). The obtained results demonstrate that SpPdp11 presents specific gene encoding proteins for gut colonization, bile salt resistance, and gut pathogen adhesion inhibition, which helps to explain some of the demonstrated in vitro and in vivo properties of this probiotic [24]. In addition, in this same study, it was demonstrated that pathogenic strains of the same species of SpPdp11 did not present such genes.

## 4. Fish Activities Modulated by SpPdp11 

The main results and studies available so far about fish activities, which are modulated by SpPdp11 administration, are now considered, and summarized in Table 1.

### 4.1. Immunity

In fish, innate and adaptive immunity are commonly divided into three components: cellular components, humoral parameters, and the epithelial/mucosal barrier [25,26]. Generally, pathogens are primarily blocked by the fish physical barriers (mucosal sites). Mucosal sites interfere with pathogens by either trapping them or through the action of several antimicrobial factors (lectins, lysozyme, antibacterial peptides, immunoglobulins, etc.), which aim to directly eliminate the infectious agent. If the pathogen is able to penetrate through the physical barriers, the cellular and humoral machinery of the immune system is triggered [27]. 

In fish, the innate immune response is essential in combating pathogens because of the limitations of the adaptive immune system. However, every component of the immune system has its own value and the final combination of them will lead to a satisfactory immune response [28]. Several studies have demonstrated that the dietary administration of different probiotics stimulate both the innate and the adaptive immune mechanisms of fish [29,30,31,32,33,34,35]. In this context, the effects of dietary administration of SpPdp11on the different components of the innate and adaptive immune system have also been evaluated.

Regarding the effects of dietary administration of SpPdp11 in cellular innate immunity, several in vivo studies have demonstrated the immunomodulatory effect of SpPdp11. Gilthead seabream specimens fed for two weeks on a SpPdp11 supplemented diet showed an increased phagocytic activity of head kidney leucocytes [29], one of the most important cellular response of leucocytes in the innate immunity [31,32]. Curiously, the respiratory burst of such leucocytes remained unaffected, in head kidney leucocytes isolated from fish fed the commercial diet without SpPdp11 [29]. Later on, a study was conducted using dietary encapsulated SpPdp11 in calcium alginate beads to improve its viability during the passage through the intestinal tract [36]. Interestingly, the obtained results corroborated that the encapsulated SpPdp11 had no immunostimulant effects on any of the tested head kidney leucocyte activities of gilthead seabream, such as peroxidase, respiratory burst, and phagocytic activity [37]. This result suggested that the probiotic needs to interact directly with the gut mucosa to modulate the cellular systemic immunity.

Concerning Senegalese sole, the oral administration of live SpPdp11 for 60 days significantly increased respiratory burst activity of head–kidney leucocytes [43]. It was considered also appropriate to test if inactivated and live SpPdp11 showed similar probiotic properties or not. In fact, inactivated probiotic preparations are also considered as an interesting alternative to the use of live probiotics, which could potentially cause safety problems in open aquatic environments due to the possibility of acquiring antibiotic resistance and virulent genes [56,57]. However, heat-inactivated bacterial cells of SpPdp11 probiotic tested in vitro did not exhibit significant or immunostimulatory influence on cellular innate immune parameters, such as peroxidase content or respiratory burst activity of gilthead seabream head–kidney leucocytes [58], whereas only live cells of SpPdp11 exerted immunostimulant effects on cellular immunity in both studied fish species [38,58].

Humoral factors may be cellular receptors or molecules that are soluble in plasma and other body fluids, such as skin mucus [59]. Regarding the immune factors present in blood, the administration of dietary alginate encapsulated SpPdp11 to gilthead seabream had immunostimulatory effects on immunoglobulin M (IgM) levels and peroxidase activity [37]. In Senegalese sole, supplementation of heat inactivated SpPdp11 also increased natural hemolytic complement and serum peroxidase activities, reaching the highest values after three and four weeks of administration [58].

The mucus of fish forms a thin physical, chemical, and biological barrier that contains several humoral components that play a key role in the innate response preventing the entry of pathogens, such as lectins, pentraxins, lysozymes, complement proteins, antibacterial peptides, or IgM [60,61]. In fact, skin mucus is currently receiving a lot of attention for determining immunity related proteins and enzymes. Several studies have addressed the effect of SpPdp11 supplemented diet on mucosal immunity by studying the effects on skin mucus. For instance, IgM levels, protease, and peroxidase activity were significantly increased in gilthead seabream skin mucus while, antiprotease remained unaffected after four weeks of administration of live cells of SpPdp11 [62].

The effects of this probiotic on immunity have also been studied by determining the effects on gene expression. In this sense, significant increases were detected in the level of mRNA of gilthead seabream head–kidney leucocytes for major histocompatibility complex II*α* (*mhcIIα*) and T-cell receptor*β* (*tcrβ*) after four weeks of administering live encapsulated SpPdp11 [37].

### 4.2. Stress 

The aquaculture environment results in a continuous exposure of fish to stress, which has deleterious effects on fish physiology [63,64]. In the last decade, several experiments have demonstrated beneficial effects of administering different probiotics in stressful situations [65,66,67]. The aim of such studies was to demonstrate if probiotic administration could have any anti-stress effect on fish or mitigate the negative impact on fish of different stressful situations. In this sense, the use of the probiotic SpPdp11 to modulate the stress response has also been evaluated.

High stocking density (HSD) condition is a chronic stressor that activates the stress axis producing high level of cortisol in fish, such as gilthead seabream [68]. Regarding this stressor, juvenile gilthead seabream specimens farmed under HSD (30 kg·m^−3^) and fed a diet supplemented with SpPdp11 showed lower levels of plasma cortisol and an improved stress tolerance than those fish under HSD receiving a control diet [41]. Another study showed that the administration of dietary live SpPdp11 for four weeks in gilthead seabream under HSD (20 kg·m^−3^) upregulated pro-inflammatory gene cytokines, such as *interleukin 1 beta* or *interleukin 6*, in comparison to the control group (5 kg·m^−3^) [44]. In addition, the same fish receiving the probiotic diet also showed an increase in cellular peroxidase and respiratory burst activity. It could be interesting to deepen research into this topic because stress can cause immunodepression and a higher susceptibility to infectious diseases in fish [45].

Furthermore, skin mucus proteome profile of gilthead seabream exposed to HSD stress (20 kg·m^−3^) after dietary SpPdp11 intake demonstrated that many proteins involved in immune processes, such as lysozyme, complement C3, natural killer cell enhancing factor, and non-specific cytotoxic cell receptor protein1 were enhanced in comparison to the values determined in fish from the control groups (5 kg·m^−3^). Perhaps, the most important result of this study was to demonstrate a consistency between lysozyme protein and lysozyme mRNA levels in skin mucus of fish fed SpPdp11 [46]. This last result corroborates a very important and local immune response—that the lysozyme present in skin mucus was synthesized by immune cells present in fish skin.

A marked reduction in the number of transcripts encoding proteins, such as G-lysozyme and heat shock protein 70 (*hsp70*) associated with high plasma levels of cortisol have been reported in Senegalese sole specimens farmed under HSD [69]. In this context, a study in Senegalese sole specimens reported that receiving a control diet and farmed under HSD an upregulation of genes related to immunological responses and it was associated with a microbial infection [47]. On the contrary, fish also farmed under HSD, but fed a diet supplemented with the probiotic showed neither upregulation of these genes nor microbial infection and the expression of the genes was very similar to that detected in fish farmed under normal stock density [45].

Furthermore, in another study, genes implicated in the response to stress, such as CCAAT/enhancer binding protein beta (*cebpb*) and several heat shock proteins (*hsp70*, *hsp90aa*, and *hsp90ab*) and in immunity including haptoglobin (*hp*), non-specific cytotoxic cell receptor protein 1 (*ncrp1*), hepcidin 1 (*hamp1*), leucocyte cell-derived chemotaxin (*lect22*), and tumor necrosis factor-alpha-induced protein 9 (*tnfαip9*) were enhanced by SpPdp11 [48]. 

All of these findings taken together support the potential use of SpPdp11 as a functional feed supplement with anti-stress properties in both fish species.

### 4.3. Disease Resistance

The sudden outbreaks of diseases and the mortality associated with them continue to be one of the major setbacks to the aquaculture industry, because of both economic losses and animal welfare [70]. In the last decades, it has been demonstrated that the use of probiotics conferred protection to fish against several pathogens including *Vibrio parahaemolyticus*, *Aeromonas salmonicida* ssp. *salmonicida*, *Flavobacterium psychrophilum*, etc. [71,72,73]. Because the probiotic SpPdp11 showed in vitro antagonistic activity against known pathogens, its ability to enhance the immune status, and improve stress tolerance of fish, it could be speculated that it might also reduce the susceptibility to microbial infections.

Different in vivo studies demonstrated that SpPdp11 oral administration significantly decreased the mortality of gilthead seabream and Senegalese sole when challenged with *L. anguillarum* DC11R2a and *V*. *harvey*i strain Lg 14/00, respectively [22,23]. After these findings, new studies (focused on the same topic) reported that administration during 60 days of a diet supplemented with SpPdp11 increased significantly the Senegalese sole head kidney leucocyte respiratory burst activity and significantly reduced mortalities when fish were challenged with *P. damselae* subsp. *piscicida*. On the other hand, another study demonstrated that the supplementation of the diet with fresh or lyophilized SpPdp11 for 60 days improved the survival rates of Senegalese sole after challenges with the former pathogenic bacteria [49]. The available results suggested that dietary SpPdp11 probiotic administration could enhance the immune status of fish and ameliorate the mortality of fish exposed to different virulent pathogens, thus, enhancing health status and protection of gilthead seabream and Senegalese sole specimens.

### 4.4. Modulation of the Microbiota

The bacterial community in the gastrointestinal tract (GI) of fish, including bacteria, yeast, viruses, archaeans, and protozoans, influences several host functions, such as digestion, immunity, protection from pathogenic organisms, and brain development [74]. The teleostean intestinal microbiota also plays an important role as a defensive barrier against infections and it regulates the expression of genes in the digestive tract related to epithelial proliferation, nutrient metabolism, and genes involved in the innate immune response [75,76]. Fish live in aqueous environments in which their mucosae are exposed to potential pathogens and when feeding; water, together with all the resident microorganisms, is taken into the GI. The GI tract is the route of nutrient uptake and any perturbation in it can be harmful to the fish. The gut microbiota plays a significant role in maintaining fish health and their balance is crucial in reducing the health-related risks factors [17], therefore new prospects for optimizing health and productivity in aquaculture systems have emerged to include insights into the farmed fish microbiota [77,78].

Different authors have reported that the intake of probiotics modified the intestinal microbiota composition towards beneficial effects to the host [12,79]. However, the effect of the probiotics on the intestinal microbiota of fish is an important aspect to consider because parameters, such as richness and biodiversity of the microbial community, can be affected. Bacterial diversity has an important role in the function of ecosystems [80], and their stability is influenced by species and functional group richness [81]. Biodiversity protects ecosystems against declines in their functionality and allows for adaptation to changing conditions, because the coexistence of many species provides a greater guarantee that some will back up a given function when others fail [82,83]. In this context, several studies have addressed the impact of SpPdp11 on fish gut microbiota, demonstrating that dietary administration of this probiotic to larval and juvenile Senegalese sole specimens usually induces slight decreases of the biodiversity [50,84], whereas in the case of juvenile gilthead seabream, this effect was not observed [37]. 

In contrast, and based on the criteria proposed by Marzorati et al. [85] to calculate the range-weighted richness of a community, the probiotic supplementation in both Senegalese sole and gilthead seabream increased the genetic variability of the intestinal microbial community [37,51], results that, according to De Schryver et al., can be considered as beneficial [86]. In addition, the dietary administration of SpPdp11 to juvenile Senegalese sole specimens resulted in higher similarity values for the predominant members of the intestinal microbiota [40] and in a higher adaptability if this community was exposed to dietary changes [50].

In a study carried out to analyze the effects of oxytetracycline on the intestinal microbiota of Senegalese sole specimens, the antibiotic induced a heavy decrease in the richness and biodiversity of the bacterial intestinal community, but these effects were lessened by the dietary administration of SpPdp11 [52]. In addition, Firmicutes such as *Lactobacillus* genus were detected only in fish receiving jointly the probiotic and the antibiotic [52]. Another interesting result was a close relationship among the intestinal microbiota of fish receiving the probiotic diet, and the expression of genes related to the anti-apoptotic effects and oxidative stress regulation, such as natural killer cell enhancement factor (*nkef*)*,* insulin growth factor β *(igf-β), hsp70,* and chaperon protein gp96 (*gp96*), conferring protection to the cells against oxidative damage, cell death, and tissue repair after injury [52]. 

Previously, it has been demonstrated that the administration of SpPdp11 to farmed Senegalese sole larvae and juvenile specimens under HSD promoted the presence of *Lactobacillus* species, such as *L. helveticus* and *L. fermentum* using denaturing gradient gel electrophoresis (DGGE) [51]. In another study, the ability of the probiotic diet to increase the predominant bands related to *Lactococcus* and *Lactobacillus* in the intestinal microbiota of gilthead seabream specimens was observed [37]. The increased presence of these bacterial groups could suggest a beneficial effect because of the capability of different *Lactobacillus* species to increase fish immunological response [87,88].

Tapia-Paniagua et al. [84] also observed the ability of the probiotic SpPdp11 to reduce the presence of *Vibrio* genus, such as the species *V. harveyi* and *V. parahaemolyticus*, and *P. damselae* subsp. *piscicida*, all of them described as pathogenic for Senegalese sole and gilthead seabream in larvae and juvenile of Senegalese sole specimens. This result could be related to the higher presence of species of the *Lactobacillus* genus, whose strains showed the ability to inhibit the adhesion to intestinal mucus and activity against *Vibrio* species [89,90].

In the study carried out by Tapia-Paniagua et al. [45] with Senegalese sole specimens farmed under HSD, an increase in the number of goblet cells was observed in the intestine of fish fed the probiotic diet in comparison with the intestine of specimens receiving the control diet without the probiotic. In fish fed the probiotic diet, the number of goblet cells was correlated with the presence of microbes only present in the intestinal microbiota [45].

Saenz de Rodrigáñez et al. and García de la Banda et al. [38,39] reported lower lipid droplet (LD) levels inside enterocytes and hepatocytes of Senegalese sole specimens fed diet supplemented with SpPdp11, in comparison with fish fed a control diet. Tapia-Paniagua et al. [84] demonstrated a correlation between the lower levels of LD and the presence of microorganisms, such as *S. putrefaciens* and strains of *Vibrio*, detected in fish fed the probiotic diet but not in those receiving the control diet. 

It was suggested that functionality of such cells should be better than that of the control group and this could contribute to the higher growth observed in several studies [49]. Furthermore, it was corroborated that dietary SpPdp11 administration can improve stress tolerance, not only by regulating the expression of several important immune genes, but also by changing the intestinal microbiota diversity, associated with an increase in the number of goblet cells in fish fed the probiotic diet [45]. 

All of these results suggest that the dietary administration of this probiotic could exert a beneficial effect on the intestinal bacterial community and play a key role in the maintenance of fish homoeostasis, resulting in an effective tool to improve sole larviculture.

### 4.5. Nutrition and Growth

The microbial modulation performed by probiotics previously discussed may also help to enhance the nutritional status and growth of the host [12]. Different studies have demonstrated that probiotic administration improves feed conversion, growth rates, and weight gain of fish [14,65]. Regarding SpPdp11 supplementation, results have shown that juvenile Senegalese sole and gilthead seabream specimens fed SpPdp11-enriched diets had a significantly higher growth performance [38,39,41] and even in the case of Senegalese sole, it induced an increase in the muscle protein content compared to the specimens fed non-probiotic diets [38]. 

Recently, it has also been demonstrated that SpPdp11 administration from the first exogenous feeding resulted in beneficial effects on Senegalese sole larval development, given that specimens fed this diet exhibited higher and less dispersed weight and size [39,42,48]. Homogeneous growth is particularly important in the aquaculture industry since its disruption can lead to dominance from bigger individuals due to social hierarchy, and because of this, to a decrease in fish production [91].

Moreover, regarding enzymatic activities involved in nutrition, Senegalese sole specimens receiving diets supplemented with SpPdp11 for 60 days increased leucine aminopeptidase activity in the distal intestine [38]. This enzyme has been used as an indicator of the enterocyte maturation and differentiation through the ontogenetic development of marine fish [92]. They are also related to the nutritional status of the animal and the level of maturation of the enterocytes, since their activities seem to reflect an adequate digestion and/or absorption of the ingested feed [93].

These results lead to the hypothesis that SpPdp11 enhances the functionality of the intestine, with the subsequent more efficiently feed utilization. Additionally, lyophilized cells of SpPdp11 significantly increased liver linolenic and linoleic acids levels [39]. Linoleic acid is involved in the synthesis of important molecules, such as triglycerides and lipoproteins [94]. This is highly relevant taking into account that fish have a low ability to convert linoleic acids into arachidonic, eicosapentaenoic, and docosahexaenoic acids, which are important to optimize feed utilization [95,96]. Another study documented the changes in the expression of a set of genes involved in central metabolic functions in Senegalese sole larvae including genes coding for proteases, such as carboxypeptidase A1 (*cpa1*), trypsinogen (*tryp1*), cathepsin Z (*ctsz*), and proteasome 26S non-ATPase subunit3 (*pmsd3*). This gene expression increase could lead to a better development and functionality of the gastrointestinal tract [48].

Several studies have been carried out to evaluate the effects of dietary administration of SpPdp11 using *Artemia* metanauplii as live vector in a co-feeding regime. When a pulse from 10 to 86 days after hatching (dah) of the probiotic was applied, it significantly promoted early metamorphosis, protein content, docosahexaenoic acid/eicosapentaenoic acid (DHA/EPA) ratios, and less size variability was obtained from metamorphosis until the end of weaning [47]. When a shorter pulse (10–30 dah) of SpPdp11 was administered, the results obtained showed a significant increase of total protein and lipid content in SpPdp11-enriched *Artemia*, whereas the same pattern of enzymatic activities was observed in control and experimental groups. However, at day 30, alkaline protease and chymotrypsin activities were significantly higher in larvae fed SpPdp11-enriched *Artemia*. Afterwards, at day 56 dah, when weaning started, no difference was found between both experimental groups [42].

Live prey, such as *Artemia*, is widely used in larviculture of Senegalese sole. However, it is naturally deficient of unsaturated acids, which are very important for the normal development and production of healthy fingerlings. It has been reported that the lipid composition of SpPdp11-enriched *Artemia* metanauplii revealed important differences compared to control *Artemia* metanauplii. In particular, a significant increase in total fatty acid contents, specifically n-3 highly unsaturated fatty acids (HUFA) levels, were observed in SpPdp11-enriched *Artemia* [53]. This ability of SpPdp11 strain to produce n-3 HUFA, improved Senegalese sole larval and fry growth, and generated changes in total lipid contents and fatty acid profiles persisting along the first stages of larval development. Considering these results, the probiotic SpPdp11 might be used as an effective tool for fish marine larviculture optimization in terms of growth and body composition.

### 4.6. Other Activities

Oxidation is a vital process in aerobic organisms, which leads to the formation of reactive oxygen species (ROS). Antioxidants can protect from free radicals, and have been used in human dietary supplements to boost health and reduce the risk of disease. For a long time, fish have been treated with chemical compounds to deal with disease or can even be exposed to them in the aquatic environment [54]. Such exposure generally induces an excessive production of free radicals being harmful for the fish. Considering this, a study evaluated the effect of the administration of the probiotics SpPdp11, *Bacillus sp.*, and date palm fruit extracts (as immunostimulants and prebiotics) for two and four weeks on the expression of the main antioxidant enzyme defense genes in gut, skin, and gill of gilthead seabream [54]. The reason to combine SpPdp11 with *Bacillus sp.* and date palm fruit extract was due to the hypothesis that a combination of different probiotic strains or prebiotics might be more effective [54]. Their results demonstrated a synergistic effect of SpPdp11 together with *Bacillus sp.* and diet palm extracts supplemented diet enhancing the expression of mucosal antioxidant genes, such as glutathione reductase, catalase, and superoxide dismutase, primarily in the gill and skin, especially after four weeks of administration. Afterwards, the ability of SpPdp11 administered through the diet to upregulate the transcription of the gene encoding glutathione peroxidase in head kidney of Senegalese sole was reported [50]. Thus, such findings lead to the hypothesis that dietary probiotics in combination with prebiotics could potentially enhance gilthead seabream mucosae enzyme antioxidant defenses contributing to the health status of fish. Furthermore, SpPdp11 administration resulted in the upregulation of the transcription of genes encoding for glutathione peroxidase (*GPx*) and *HSP70*, indicating a potential protective effect of SpPdp11 against oxidative stress in Senegalese sole [50].

A recent study has suggested that SpPdp11 could be powerful in alleviating intestinal dysfunction caused by skin wounds. In this study, the crosstalk between skin wounds and the intestinal barrier together with the administration of SpPdp11 as a prophylactic tool were examined. Gilthead seabream were fed either a control or supplemented diet after being injured. Control fish showed disordered enterocyte nucleus disposition, a higher intense infiltration of mixed leucocytes, and a thicker lamina propria, while the fish fed SpPdp11 did not show any of these pathologies. Furthermore, Spdp11 dietary administration downregulated the expression of pro-inflammatory cytokines while increasing anti-inflammatory cytokines [55]. Thus, the use of SpPdp11 as a preventive measure to treat alterations in the intestine of gilthead seabream could be further studied.

## 5. Conclusions

Sustainable aquaculture is the key to develop this continuously growing industry. In this context, several strategies are in the focus of attention of many researchers, such as the development of new immunostimulants, probiotics, prebiotics, or symbiotics. The results obtained from all the studies carried out until present have shown that oral administration of SpPdp11 (viable or non-viable) as a probiotic in the culture of gilthead seabream and Senegalese sole has several stimulatory effects and can be highly effective by contributing to host metabolism, nutrition, growth, immune response, stress response, disease resistance, and fish survival. It is important to mention that most of the functions modulated by SpPdp11 were mainly enhanced after three or four weeks of administration, leading to the conclusion that longer periods of administration are not necessary and prophylactic use might be enough to achieve the desirable outcomes. Moreover, the administration route more adequate is dietary supplementation and the administration dose has been established to be effective in the range of 10^8^ cfu·g^−1^–10^9^ cfu·g^−1^ for gilthead seabream and Senegalese juvenile stages and lesser (2.5 × 10^7^ cfu·mL^−1^) for Senegalese sole larval stage. 

## 6. Future Perspectives

Several aspects might be considered for future studies, such as other administration routes (e.g., directly adding the probiotic to the water column) when the administration via feeding (pellet) has some limitations (for example, during early larval stages). Until present, there are no studies with SpPdp11 directly added to the rearing water, even though it is the only method that is applicable for all fish stages. It would be interesting to apply such method and to test the possible immune and nutritional stimulatory effects of SpPdp11 in gilthead seabream larval stages. 

Moreover, studies using a combination with prebiotics and/or other probiotic species, such as those by Esteban et al. (2014) [94], could be beneficial to boost SpPdp11 properties. Furthermore, the mechanisms underlying the beneficial effects of probiotics in aquatic systems are rarely studied in depth. Further studies are needed to elucidate these mechanisms and potential harmful effects.

The efficacy of this probiotic in microbial infections could also be studied, taking into account the promising results obtained in fish infected with pathogenic bacteria. On another note, next-generation sequencing (NGS) technologies are more accessible to researchers. The metagenomic profile of fish gut, skin, and gill microbiota should be further investigated to elucidate the SpPdp11 mode of action. It has been suggested that bacterial metabolites play a key role in the orchestration of the host immune response, specifically by the recognition of microbial patterns by the innate immune system, which triggers a signaling cascade downstream [96]. A more comprehensive understanding of how microbiota-derived metabolites shape the fish immune system would be essential to know the action mode of the probiotic SpPdp11.

Since SpPdp11 was originally isolated from gilthead seabream and has had many beneficial effects on other species, such as Senegalese sole, it would be also interesting to test other economically important cultured species in the Mediterranean, such as sea bass (*Dicentrarchus labrax*) or turbot (*Psetta maxima*), among others. Perhaps, more prophylactic and therapeutic uses of this probiotic could be demonstrated in the near future (such as anti-viral, anti-parasitic or anti-inflammatory properties, or if the probiotic is able to ameliorate heavy metal fish exposure), with the final aim to collaborate for the improvement of the farmed fish industry.

## Figures and Tables

**Table 1:** Summary of all results and major outcomes from all studies performed using SpPdp11.

Species	Concentration	Route of Administration	Fish Stage, Average Weight	Experiment Duration (days)	Major Outcomes	Reference
**Senegalese sole**	10^8^ cfu g^−1^	lyophilized, diet supplementation (dry pellet)	Juvenile, 15–20 g	15	Reduced mortality after *Vibrio harveyi* challenge	[22]
**Gilthead seabream**	10^8^ cfu g^−1^	heat inactivated, diet supplemented (dry pellet)	Juvenile, 65 g	28	Improved cellular and humoral immunity	[29]
**Senegalese sole**	10^9^ cfu g^−1^	lyophilized, diet supplementation (dry pellet)	Juvenile, 10–15 g	60	Modulation of intestinal microbiota. No lipid droplets in enterocytes.	[38]
**Senegalese sole**	10^9^ cfu g^−1^	encapsulated in calcium alginate beads	Juvenile, 26.7 ± 4.6 g	60	Improved growth rate and survival after *Photobacterium damsela*e subsp. *Piscicida*	[39]
**Senegalese sole**	10^9^ cfu g^−1^	fresh and lyophilized cells added to the pellet	Juvenile, 26.7 ± 4.6 g	60	Modulation of intestinal microbiota	[40]
**Gilthead seabream**	10^9^ cfu g^−1^	live cells, directly sprayed in pellet	Juvenile, 38.28 ± 0.81 g	116	Improved growth performance and stress tolerance under high stocking densities	[41]
**Senegalese sole**	2.5 × 10^7^ cfu mL^−1^	bioencapsulated in live vector (*Artemia*)	Larvae, 10–30 dph	20	Modulation of gut microbiota. Better growth performance and body composition	[42]
**Gilthead seabream**	10^8^ cfu g^−1^	encapsulated in calcium alginate beads	Juvenile, 41.6 g	28	Improved humoral immunity. Up-regulation in immune related genes. Modulation of intestinal microbiota	[37]
**Gilthead seabream**	10^8^ cfu g−1	lyophilized, diet supplementation (dry pellet)	Juvenile, 15–20 g	15	Reduced mortality after *L. anguillarum* challenge	[23]
**Senegalese sole**	10^9^ cfu g^−1^	lyophilized, diet supplementation (dry pellet)	Juvenile, 10–17 g	60	Improved cellular immunity. Mortality reduced after *Photobacterium damselae* subsp. *Piscicida* challenge	[43]
**Gilthead seabream**	10^8^ cfu g−1	fresh cells added to the diet (dry pellet)	Juvenile, -	28	Improved cellular and humoral immunity and gene expression profile of proinflammatory cytokines under stress	[44]
**Senegalese sole**	10^9^ cfu g^−1^	live cells, directly sprayed in pellet	Juvenile, 14.6 ± 0.7 g	30	Modulation of the intestinal microbiota under stress	[45]
**Gilthead seabream**	10^8^ cfu g^−1^	lyophilized, diet supplementation (dry pellet)	Juvenile, 104.2 g	30	Positive proteomic changes in skin mucus under stress	[46]
**Senegalese sole**	2.5 × 10^7^ cfu mL^−1^	bioencapsulated in live vector (*Artemia*)	Larvae, 10–86 dph	76	Modulation of gut microbiota and increased DHA/EPA ratios. Enhace growth in length and weight	[47]
**Senegalese sole**	2.5 × 10^7^ cfu mL^−1^	bioencapsulated in live vector (*Artemia*)	Larvae, 2–73 dph	71	Beneficial effects on larval development. Up-regulation of genes related to growth and immunity	[48]
**Senegalese sole**	10^9^ cfu g^−1^	fresh and lyophilized cells added to the pellet	Juvenile, 23.4 ± 0.3 g	60	Higher growth rates with fresh cells. Both fresh and lyophilized cells conferred protection against *Photobacterium damselae* subsp. *Piscicida*	[49]
**Senegalese sole**	10^9^ cfu g^−1^	live cells, directly sprayed in pellet	Juvenile, 26.7 ± 4.6 g	21	Higher adaptability to dietary changes in the intestinal microbiota and potential protective effect against oxidative stress	[50]
**Senegalese sole**	10^9^ cfu g^−1^	lyophilized, diet supplementation (dry pellet)	Juvenile, 26.7 ± 4.6 g	69	Modulation of intestinal microbiota	[51]
**Senegalese sole**	10^9^ cfu g^−1^	-	Juvenile, 14.57 ± 0.71 g	10	Administration of OTC and SpPdp11 increases the transcription of genes related to antiapoptotic effects and oxidative stress regulation.	[52]
**Senegalese sole**	2.5 × 10^7^ cfu mL^−1^	bioencapsulated in live vector (*Artemia*)	Larvae, 10–30 dph	21	Increased total lipids (n-3 HUFA) and higher growth performance	[53]
**Gilthead seabream**	10^9^ cfu g^−1^	live cells, directly sprayed in pellet	Juvenile, 12.5 ± 2.2 g	28	Improved antioxidant activity mainly in gills and skin	[54]
**Gilthead seabream**	10^9^ cfu g^−1^	fresh cells added to the diet (dry pellet)	Juvenile, 21.81 ± 0.87 g	30	Beneficial effects regarding the negative effects in intestinal histology, depressed expression of pro-inflammatory and increased expression of anti-inflammatory cytokines after wounding	[55]
